# U.S. vs. Foreign Nativity and 10-Year Trajectories of Mental Health after Traumatic Brain Injury: A Model Systems Study

**DOI:** 10.3390/jcm12030867

**Published:** 2023-01-21

**Authors:** Chimdindu Ohayagha, Kritzia Merced, Paul B. Perrin, Juan Carlos Arango-Lasprilla, Daniel W. Klyce, Shawn C. T. Jones

**Affiliations:** 1 Department of Psychology, Virginia Commonwealth University, Richmond, VA 23284, USA; 2 Central Virginia Veterans Affairs Health Care System, Richmond, VA 23249, USA; 3 School of Data Science and Department of Psychology, University of Virginia, Charlottsville, VA 23294, USA; 4 Sheltering Arms Institute, Richmond, VA 23233, USA

**Keywords:** traumatic brain injury, nativity, disparities, mental health outcomes

## Abstract

Background: Previous research has found racial and ethnic disparities in life satisfaction, depression, and anxiety after traumatic brain injury (TBI). However, limited studies have examined differences in these variables between U.S.- and foreign-born individuals with TBI. The purpose of this study was to examine whether differences exist in mental health outcomes between U.S.- and foreign-born individuals with TBI at 1, 2, 5, and 10 years after injury, as well as examine whether demographic and injury-related characteristics account for these differences. Method: Participants were 8289 individuals with TBI who identified as U.S.-born and 944 who identified as born outside the U.S. in the TBI Model Systems study. Participants completed measures of mental health outcomes at 1, 2, 5, and 10 years after injury. Results: Foreign-born individuals with TBI had comparable levels of depression and anxiety trajectories to U.S.-born individuals, yet higher life satisfaction trajectories, even after controlling for demographic and injury-related variables. Conclusion: Rehabilitation professionals should consider in their clinical work the mechanisms that likely influence mental health outcomes among foreign-born individuals, including family-based values that increase resilience, as well as the possible under-reporting of mental health symptoms along the lines of cultural norms.

## 1. Introduction

Approximately 69 million people sustain a traumatic brain injury (TBI) every year [1], often resulting in lifelong disability [2]. In 2014, there were approximately 2.87 million TBI-related emergency department visits, hospitalizations, and deaths in the U.S. [3], representing a 53% increase from 2006. TBI impacts on the individual, their families, and the health systems providing post-injury care. People with TBI may experience changes in physical, social, cognitive, and/or psychological functioning [3] and are at risk for long-term psychiatric symptoms [4], with anxiety and depressive disorders representing the most common mental-health concerns [5].

Individuals with TBI from racial and ethnic minority backgrounds report worse mental health across depression, anxiety, and life satisfaction when compared to white people [6,7,8]. Seel et al. [6] found that Black people with TBI reported greater depression symptoms compared to white people, and Arango-Lasprilla and colleagues [7] found that Black people with TBI reported lower life satisfaction compared to white and Asian people. Regarding other domains of mental health, Black people reported greater PTSD symptomatology at 12 months post-injury compared to white people [9]. Jimenez et al. [8] showed that Latinx children reported larger reductions in life satisfaction relative to white children three years post-injury. However, Hart et al. (2005) [10] found that Black and white groups who had comparable pre-injury characteristics reported similar outcomes, with both groups showing increases in depression symptoms and lower satisfaction with life, suggesting no differences over time in life satisfaction after controlling for demographics.

There remains a large gap in research examining cultural and U.S.- vs. foreign-nativity factors that can influence rehabilitation outcomes following TBI (e.g., social stigma, cultural isolation, acculturation), though the emerging studies to date have focused nearly exclusively on native language. About 85% of the U.S. foreign-born population speaks a language other than English at home [11]. Lequerica et al. [12] found that that individuals with TBI who did not speak English were rated worse on functional communication outcomes at inpatient rehabilitation discharge compared to individuals whose primary language was English. Research also shows that individuals with limited English proficiency are often misdiagnosed and incorrectly treated by healthcare systems [13]. In addition to demographic and socioeconomic contributors to racial/ethnic disparities, research has documented disparities concerning post-injury hospitalization [14], rehabilitation referrals [15], and post-hospital discharge location [16].

Given that racial and ethnic disparities in psychological adjustment are present after TBI, exploring cultural factors impacting post-TBI mental health and functioning is critical [17,18]. The current study examined whether differences exist in mental health outcomes between U.S.- vs. foreign-born individuals with TBI at 1, 2, 5, and 10 years after injury. The study then examined whether demographic and injury-related characteristics contributed to these differences.

## 2. Materials and Methods

### 2.1. Participants

Participants were enrolled in the TBI Model System (TBIMS) study. TBIMS is a multicenter longitudinal study that comprises of 16 level 1 trauma centers and 3 longitudinal follow-up centers. Participants of the TBIMS program receive initial care in an emergency department, followed by management of acute neurotrauma, comprehensive inpatient rehabilitation, and long-term outpatient services. To be included in the parent study, participants must (a) be 16 years of age or older at the time of injury, (b) have a medically diagnosed TBI (e.g., mild complicated, moderate, or severe TBI), (c) have either a Glasgow Coma Scale (GCS) score of ≤12 upon emergency admission, >24-h duration of post-traumatic amnesia (PTA), loss of consciousness (LOC) >30 min, or evidence of intracranial trauma on neuroimaging, (d) be admitted to a TBIMS acute care hospital within 72 h of injury, and (e) complete inpatient rehabilitation services within a TBIMS center. For the current study, we used the variable of “country of birth” to differentiate U.S.- vs. foreign-born groups. A participant was designated as “U.S.-born” if they were born in the U.S. and as “foreign-born” if they were born outside of the U.S. but were undergoing acute rehabilitation (and were therefore extremely likely to have sustained their TBI and enrolled in the current study) while in the U.S. The TBIMS database does not collect data on the specific country of birth.

Participants with recorded data of (1) their country of birth and (2) the outcome variables from at least one follow-up point were included. The inclusion of the predictor (country of birth) and the mental-health dependent variables (measures of depression, life satisfaction, and anxiety) resulted in a final sample size of 9233 participants. Of these, 8289 identified as U.S.-born and 944 identified as born outside the U.S. The two groups were compared on demographics using analyses of variance (ANOVAs) for continuous variables and 𝜒^2^ tests for categorical variables (Table 1).

### 2.2. Procedure 

Study procedures were approved by the individual Institutional Review Boards for each TBIMS center. Informed consent was provided by either the participant or when appropriate their legal guardian, family member, or their proxy. All data collection was completed through the review of medical records and interviews with participants by highly trained research staff. Follow-up data were collected via telephone interviews, and participants also had the option to choose an in-person interview or complete a self-administered questionnaire. The vast majority of data collections occurred in English, although some TBIMS sites were able to administer questionnaires in Spanish if their staff had that capacity and a participant required it. Data collection language is not systematically documented in the database.

### 2.3. Measures

Satisfaction with Life Scale (SWLS). The SWLS is a 5-item measure of life satisfaction [19]. Item responses are on a 7-point Likert-type scale ranging from strongly disagree (1) to strongly agree (7). Scores range from 5 to 35 with higher scores representing greater life satisfaction. Extreme satisfaction and dissatisfaction with life are represented by scores ranging from 26 to 30 and 5 to 9, respectively [20]. The scale has demonstrated strong psychometric properties [20] and is well-validated and reliable among individuals with TBI [21,22].

Patient Health Questionnaire-9 (PHQ-9). The PHQ-9 is a 9-item measure of depression based on diagnostic criteria [23]. Respondents rate the frequency and severity of depressive symptoms over the last two weeks [24]. Each item is on a Likert-type scale from 0–3 where 0 indicates “not at all”, 1 “several days”, 2 “more than half the days”, and 3 “nearly every day”. Total scores range from 0 to 27, with higher scores indicating higher levels of depression symptomatology. The PHQ-9 has a sensitivity of 88% and a specificity of 88% in the diagnosis of major depression when the cut-off score of ≥10 is used [23]. In two separate PHQ-9 studies, the internal consistency was good (α = 0.89 and 0.86) [23].

Generalized Anxiety Disorder-7 (GAD-7). The GAD-7 is a 7-item measure of generalized anxiety based on diagnostic criteria [25]. Respondents rate the severity of symptoms over the last two weeks. Each item is on a Likert-type scale from 0–3 where 0 indicates “not at all”, 1 “several days”, 2 “more than half the days”, and 3 “nearly every day”. Total scores range from 0 to 21, with higher scores indicating higher anxiety. The GAD-7 scale has good test-retest reliability and excellent internal consistency [25] and has been used extensively among individuals with TBI [26].

### 2.4. Data Analysis

Descriptive statistics (i.e., means, standard deviations) of outcome variables were generated using SPSS 27. Because of the high potential of missing data in longitudinal data collection, hierarchical linear modeling (HLM) with full information maximum likelihood (FIML) estimation was conducted to include participants with missing data.

Preliminary Curvature Analyses. An initial set of three HLMs was run with only the intercept and (a) time, (b) addition of time × time, and (c) addition of time × time × time as fixed effects’ predictors in order to determine whether a linear (e.g., straight line), quadradic (e.g., U-shaped), or cubic (e.g., S-shaped) model, respectively, most accurately reflected each type of outcome data over time (i.e., three sets of HLMs, one for each outcome). The −2 Log likelihood (−2 LL) values were compared for each successive model with a critical 𝜒^2^ value for significant difference at α = 0.05 being a ≥3.841 drop from the previous model (at 1 degree of freedom).

Primary Set 1. The next set of HLMs assessed differences in each of the three mental health outcomes over time in the U.S.- and foreign-born groups. Follow-up HLMs within each outcome incorporated interaction terms between time and nativity to determine if these differences in mental health outcomes, only when present, occurred differentially as a function of time. 

Primary Set 2. For the second primary set, when nativity-based differences on an outcome were present, this same series of analyses was conducted with the inclusion of the following demographic and injury-related variables as covariates to determine whether these variables accounted for the effects of nativity in the first analysis: violence as a cause of injury (1 = violent, 0 = not violent), GCS, PTA, age, sex, years of education, employment status at injury (1 = competitively employed, 0 = not competitively employed), marital status at injury (1 = married, 0 = not married), annual earnings, and health insurance status (1 = private insurance, 0 = public or no insurance). Similarly, if appropriate, follow-up HLMs incorporated interaction terms between time and nativity, with the same covariates included, to determine if these differences in mental health outcomes occurred differentially as a function of time even after co-varying for possible confounds.

## 3. Results

### 3.1. Satisfaction with Life Scale (SWLS)

Descriptive and Curvature Analyses. Means and standard deviations (SDs) for the SWLS at each time point were calculated (Table 2). The average participant score in the sample fell into the slightly satisfied range [20], with a slight upward movement for the first 5 years, and scores remained relatively stable from the 5- to 10-year time point. A comparison of curvature models suggested that a quadratic, or U-shaped, trend best fit the SWLS trajectories (Table 3).

**Figure 1:**
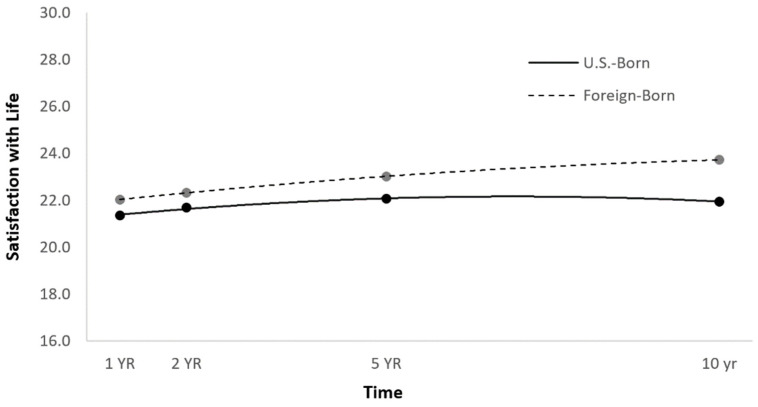
Main effect of nativity on SWLS trajectories.

Primary Set 1. The first set of HLMs examined whether quadratic SWLS trajectories could be predicted by nativity. All statistically significant and non-significant fixed effects from the HLM and their b-weights and *p*-values appear in Table 4. There was a significant main effect of nativity on SWLS trajectories, suggesting that foreign-born individuals with TBI had higher life satisfaction scores over time than those born in the U.S. (Figure 1).

However, there was no significant time^2^ × nativity effect (Table 4), suggesting that SWLS trajectories did not change differentially over time as a function of nativity.

Primary Set 2. The HLM with demographic and injury-related covariates added to the model found that the effect of nativity on SWLS trajectories remained, even after controlling for possible confounds (Table 4). Because the original time^2^ × nativity effect had not been significant, no respective follow-up model was run with the addition of covariates.

### 3.2. Depressive Symptoms (PHQ-9)

Descriptive and Curvature Analyses. The average participant PHQ-9 score in the sample fell into the mild depressive symptom range over time [23] which remained relatively stable at each successive time point (Table 2). A comparison of curvature models suggested that a linear, or straight-line, trend best fit PHQ-9 trajectories over time (Table 3).

Primary Set 1. There was no significant main effect of nativity status on PHQ-9 trajectories, suggesting that foreign-born individuals with TBI had statistically equivalent depression scores over time relative to individuals born in the US. As a result, no additional PHQ-9 models were run attempting to account for effects that were not found. 

### 3.3. Generalized Anxiety Disorder (GAD-7)

Descriptive and Curvature Analyses. The average participant GAD-7 score in the sample fell into the minimal anxiety symptom range over time [25] which remained relatively stable at each successive time point (Table 2). A comparison of curvature models suggested that a linear, or straight-line, trend best fit the GAD-7 trajectories over time (Table 3).

Primary Set 1. There was no significant main effect of nativity status on GAD-7 trajectories, suggesting that foreign-born individuals with TBI had statistically equivalent anxiety scores over time relative to individuals born in the U.S. As a result, no follow-up GAD-7 models were run.

## 4. Discussion

The purpose of this study was to examine the influence of nativity on mental health (i.e., life satisfaction, depression, and generalized anxiety) outcomes in individuals with TBI at 1, 2, 5, and 10 years post-injury. Broadly, the average scores for life satisfaction in the sample fell into the slightly satisfied range, with a slight upward movement for the first 5 years, with scores remaining relatively stable in the 5- and 10-year time points. These findings are consistent with prior research showing that life satisfaction is generally lower among individuals with TBI in the first year after injury [27] and improves over time [28,29]. A Swedish study [30] found that participants were generally satisfied across 10 domains of life satisfaction at 15 years post-injury. Williamson et al. [31]. also found in a U.S. TBIMS sample that life satisfaction trajectories increased over 10 years post-injury across the full sample. In the current study, average participant PHQ-9 and GAD-7 scores fell into the mild depressive and anxiety symptom range over time [23] and remained relatively stable at each successive time point. The results were contrary to previous literature, where depression has been reported as changing over time [32,33,34,35].

Foreign-born people in the current study had comparable depression and anxiety to those born in the U.S. and higher life satisfaction trajectories over time, even after controlling for covariates. This finding was inconsistent with known racial and ethnic disparities in mental health after TBI [36,37], and instead adds to a burgeoning body of research on protective and resilience factors among foreign-born individuals. Conversely, it is quite consistent with research in non-TBI samples, for example showing that that foreign-born Hispanic individuals report higher life satisfaction than other racial and ethnic groups, possibly reflecting the Immigrant Paradox [38], or the notion that immigrants have better psychosocial and health outcomes than would be expected based on other indices of socioeconomic status. To fully conceptualize adjustment to TBI in foreign-born individuals, it is important to consider the contributing factors of the individual’s larger culture. Culture plays a vital role in decisions about health, long-term care service use, and rehabilitation [39]. Knowing the cultural preferences of individuals with TBI may provide a greater understanding of psychological adjustment, not only facilitating the development of culturally competent services but also increasing treatment adherence and patients’ quality of life [40]. These cultural values have often been suggested to include familism and interdependence [41].

In many countries, particularly Latinx, Asian, and African countries, family values tend to be embedded in societal structure [42,43]. As family members are the major source of support for individuals with TBI, they play a pivotal role in promoting healthy psychological adjustment throughout the rehabilitation process. Gan et al. [44] found that the level of family functioning is inversely associated with stress levels in individuals with TBI. Increased family collaboration in rehabilitation processes is linked to better TBI rehabilitation outcomes [45,46,47]. Social support, in general, has been linked to greater rehabilitation outcomes following TBI [48]; thus, close family involvement may contribute to milder depression and anxiety than might be expected in foreign-born individuals with TBI or in fact to greater life satisfaction than U.S.-born individuals with TBI. Family cohesion has been shown to be a protective factor for Asian American immigrants, with higher levels of family cohesion associated with lower rates of depressive disorders [49]. However, studies also show that greater family cultural conflict is associated with higher rates of depression and lower psychological well-being among immigrants [50,51,52]. 

Mental health among foreign-born groups in the U.S. can be difficult to assess and treat due to several factors. Negative social attitudes toward mental illness, somatically focused symptom presentation, lack of understanding of mental illness among family members or care providers, and avoidance of mental health services by patients [53] are common among foreign-born individuals. Due to the negative stigma that is generally associated with mental health issues, immigrants are less likely to disclose their emotional symptoms, and they may have more difficulty receiving treatment [54]. Culture affects the reported prevalence of mental health issues, issues with diagnosis and assessment, etiology, course of the disease, how distress can be expressed, certain coping styles and help-seeking behaviors, as well as issues with treatment [55]. As such, foreign-born individuals in the current study may also have been under-reporting their mental health issues or over-reporting their life satisfaction, in line with cultural norms.

Although in the current study, there was a profound overlap between racial and ethnic minority status and nativity (e.g., the U.S.-born sample was 74.0% white, 1.0% Asian, and 5.6% Hispanic, whereas the foreign-born sample was 25.6% white, 15.2% Asian, and 46.0% Hispanic), it is important not to conflate race and ethnicity with nativity. Because U.S. vs. foreign nativity was extremely multicollinear (statistically speaking) with race and ethnicity, and HLMs cannot handle predictors that are not dichotomous or continuous (e.g., race and ethnicity which has many non-linear levels), it was not possible to co-vary for race and ethnicity with any nuance other than a false dichotomy of white vs. other. This omission of race and ethnicity in the analyses could lead one to wonder whether the findings reflecting no nativity-based differences in anxiety and depression but higher life satisfaction in the foreign-born subsample were an artifact of an oversimplified nativity dichotomy. We would argue “no” for two reasons. First, a recent study [56] using the same analytic approach as in the current study found that relative to U.S.-born individuals with TBI, those who were foreign-born had lower motor and cognitive functioning trajectories over the 10 years after TBI, as well as greater supervision needs. As a result, the current study’s analytic strategy was sensitive enough to identify nativity-based disparities in TBI outcomes where they exist and by comparison suggested that despite the previously identified reduced functional outcome trajectories, foreign-born individuals still have comparable or better psychological adjustment to TBI than U.S.-born individuals. Second, using the TBI Model Systems database, Perrin et al. [37] found that Black and Latinx individuals had elevated depression and anxiety symptom trajectories, and Williamson et al. [31] found that Black individuals had lower life satisfaction trajectories. As a result, the current findings cannot be explained simply by a confound between race/ethnicity and nativity. Nonetheless, future research should examine TBI outcomes in more of an intersectional way that teases apart these variables (e.g., a white immigrant with limited English proficiency who is not a target of systemic racism but other types of bias/discrimination such as xenophobia). 

### 4.1. Clinical Implications

The current findings have several implications for TBI rehabilitation, particularly for foreign-born individuals with TBI. Rehabilitation professionals should consider the mechanisms that likely influence mental health outcomes among foreign-born individuals. For instance, rehabilitation clinicians may benefit from adequate training on culturally sensitive practices to increase provider awareness and knowledge of the cultural beliefs and customs of their patients, especially those involving family norms. In addition, research has shown healthcare providers encounter many challenges related to language and communication when caring for foreign-born individuals and their families [57,58]. One area for targeted attention may be assisting providers in working with medical interpreters, including gaining familiarity with best practices and gaining comfort in working with medical interpreters and foreign-born families who are non-English speakers [59]. Some important components related to best practices may include allotting more time when working with foreign-born individuals with TBI, using the therapeutic alliance as an ongoing goal and intervention in rehabilitation, and continuously exploring appropriate communication strategies centered on the family’s style and dynamics. 

### 4.2. Limitations and Future Directions

There are several limitations in the current study. First, these data were not experimental and we are unable to determine causality in the series of relationships. However, given that nativity-based differences in life satisfaction persisted after controlling for demographic and injury-related variables, we can rule out these variables as causal factors underlying this difference. Second, previous studies have shown that the TBIMS national database does not encapsulate the full U.S. TBI population [60]. Caution should be taken in generalizing the current findings to all foreign-born groups with TBI. Third, although many foreign-born individuals with TBI may share cultural values, the difference between cultural groups (e.g., African vs. Asian vs. Latinx) as well as intragroup differences (e.g., differences within African, Asian, and Latinx groups) may also highlight nuances in cultural beliefs, attitudes, and behaviors in response to rehabilitative care. Fourth, the generation level was not captured in the current sample. Future studies should examine how generational immigration differences affect rehabilitation outcomes. One major aspect of the Immigrant Paradox postulates that the second-generation individuals born in the U.S. fare worse than the first immigrant generation for major mental health issues such as depression and anxiety [61,62]. Fifth, the vast majority of questionnaires were administered in English, with some sporadic administration by TBIMS sites in Spanish if they had the capability. To our knowledge, sites did not administer questionnaires in additional languages, which could have limited the participation of linguistically diverse individuals. As a result, the study may have excluded foreign-born individuals not proficient in English. Finally, the psychometric properties of the SWLS, PHQ-9, and GAD-7, though they have been examined in the context of TBI, have never been examined differentially in TBI by either race and ethnicity or nativity. These measures have extensive cross-cultural support in general populations, but nonetheless psychometric properties differing by nativity may have played a role in the current findings. Future research should conduct invariance tests of the instruments by race and ethnicity and nativity in TBI to lend further credence to these findings. 

## 5. Conclusions

The current study examined the degree to which disparities in mental health outcomes exist between U.S.- vs. foreign-born individuals with TBI at 1, 2, 5, and 10 years after injury. The study examined whether demographic and injury-related characteristics contribute to some of these disparities. The results suggested that foreign-born individuals with TBI have comparable anxiety and depression to those born in the U.S. and in fact better life satisfaction, even after controlling for demographic- and injury-related variables. This study supports future exploration for possible cultural and acculturative factors that may better explain these differences. 

## Figures and Tables

**Table 1:** Sample characteristics.

Demographics	U.S.-Born *M* (*SD*) or *n* (%)	Foreign-Born *M* (*SD*) or *n* (%)	*p*-Value
*N*	8289	944	
Sex			*p* = 0.101
Male	5981 (72.2%)	705 (74.7%)	
Female	2307 (27.8%)	239 (25.3%)	
Age	38.43 (17.57)	40.65 (18.12)	
Race			*p* < 0.001
Black	1490 (18.0%)	85 (9.0%)	
White	6136 (74.0%)	241 (25.6%)	
Asian	79 (1.0%)	143 (15.2%)	
Hispanic	461 (5.6%)	434 (46.0%)	
Native American	53 (0.6%)	2 (0.2%)	
Other	69 (0.8%)	38 (4.0%)	
Marital status			*p* < 0.001
Married	2588 (31.2%)	367 (39.0%)	
Not married	5698 (68.8%)	574 (61.0%)	
Education	12.82 (2.59)	12.11 (4.10)	*p* < 0.001
Employment at Injury			*p* = 0.004
Employed	4753 (65.8%)	596 (70.9%)	
Not employed	2465 (34.2%)	245 (29.1%)	
Annual Earning			*p* = 0.002
<9999	815 (17.7%)	101 (18.0%)	
10,000–19,999	742 (16.1%)	122 (21.7%)	
20,000–29,999	744 (16.1%)	111 (19.8%)	
30,000–39,999	623 (13.5%)	70 (12.5%)	
40,000–49,999	478 (10.4%)	40 (7.1%)	
50,000–59,999	320 (6.9%)	33 (5.9%)	
60,000–69,999	238 (5.2%)	22 (3.9%)	
70,000–79,999	142 (3.1%)	12 (2.1%)	
80,00–89,999	108 (2.3%)	9 (1.6%)	
90,000–99,999	94 (2.0%)	5 (.9%)	
>100,000	308 (6.7%)	37 (6.6%)	
Cause of Injury			*p* = 0.048
Non-Violent	7425 (89.7%)	822 (87.6%)	
Violent	851 (10.3%)	116 (12.4%)	
Glasgow Coma Scale (GCS) Score	10.93 (4.12)	10.84 (4.11)	*p* = 0.633
Posttraumatic Amnesia (PTA; days)	22.92 (21.98)	23.47 (24.41)	*p* = 0.519

**Table 2:** Mental health variable means (SDs).

	1 Year	2 Years	5 Years	10 Years
Satisfaction With Life Scale				
Mean (*SD*)	21.42 (8.26)	21.74 (8.38)	22.13 (8.27)	22.07 (8.30)
Patient Health Questionnaire-9				
Mean (*SD*)	5.43 (5.78)	5.32 (5.80)	5.20 (5.75)	5.43 (5.89)
General Anxiety Disorder -7				
Mean (*SD*)	4.09 (5.16)	4.25 (5.25)	4.20 (5.30)	4.03 (5.05)

**Table 3:** Curvature model comparisons for SWLS, PHQ-9, and GAD-7 trajectories.

Model	−2 Log Likelihood
Satisfaction With Life Scale	
Linear	151,885.42
Quadratic	**151,868.98**
Cubic	151,867.25
Patient Health Questionnaire-9	
Linear	**56,324.34**
Quadratic	56,322.66
Cubic	56,322.13
General Anxiety Disorder-7	
Linear	**34,946.05**
Quadratic	34,943.91
Cubic	34,943.82

Note. Critical 𝜒^2^ value for significant difference at α = 0.05 is >3.841 drop from the previous model. Bolded values surpassed this threshold.

**Table 4:** Predictors of SWLS, PHQ-9, and GAD-7 trajectories.

	SWLS		PHQ-9		GAD-7	
Predictor	*b*-Weight	*p*-Value	*b*-Weight	*p*-Value	*b*-Weight	*p*-Value
Set 1: Nativity Effects						
Intercept	21.20	<0.001	5.43	<0.001	4.20	<0.001
Time	0.27	<0.001	−0.01	0.644	−0.01	0.695
Time^2^	−0.02	<0.001	-	-	-	-
** Foreign- vs. U.S.-born**	**0.89**	**<0.001**	**−0.28**	**0.295**	**−0.36**	**0.172**
Set 1: Nativity Interactions with Time						
Intercept	21.21	<0.001	-	-	-	-
Time	0.28	<0.001	-	-	-	-
Time^2^	−0.02	<0.001	-	-	-	-
Foreign- vs. U.S.-born	0.74	0.011	-	-	-	-
Time × Nativity	−0.07	0.658	-	-	-	-
**Time^2^ × Nativity**	**0.03**	**0.156**	-	-	-	-
Set 2: Nativity with Covariates			-	-	-	-
Intercept	18.13	<0.001	-	-	-	-
Time	0.20	0.019	-	-	-	-
Time^2^	−0.01	0.293	-	-	-	-
**Foreign- vs. U.S.-born**	**1.64**	**<0.001**	**-**	**-**	**-**	**-**
Violent Cause	−2.67	<0.001	-	-	-	-
GCS	0.04	0.379	-	-	-	-
PTA	−0.04	<0.001	-	-	-	-
Age	−0.06	<0.001	-	-	-	-
Male Sex	−0.05	0.885	-	-	-	-
Years of Education	0.33	<0.001	-	-	-	-
Employed at Injury	1.32	0.048	-	-	-	-
Married at Injury	1.76	<0.001	-	-	-	-
Annual Earnings	0.18	0.002	-	-	-	-
Private Insurance	1.19	<0.001	-	-	-	-

Note. Bolded predictors within each model were those focused on for interpretation based on the study’s research aims.

## Data Availability

Data are available upon request made to the corresponding author.
